# Molecular characterization of carotenoid biosynthetic genes and carotenoid accumulation in Scutellaria baicalensis Georgi

**DOI:** 10.17179/excli2014-547

**Published:** 2015-01-26

**Authors:** Pham Anh Tuan, Yeon Bok Kim, Jae Kwang Kim, Mariadhas Valan Arasu, Naif Abdullah Al-Dhabi, Sang Un Park

**Affiliations:** 1 Department of Crop Science, Chungnam National University, 99 Daehak-Ro, Yuseong-gu, Daejeon 305-764, Korea; 2 Herbal Crop Research Division, Department of Herbal Crop Research, Bisanro 92, Eumseong, Chungbuk, 369-873, Korea; 3 Division of Life Sciences, College of Life Sciences and Bioengineering, Incheon National University, Incheon 406-772, Korea; 4 Department of Botany and Microbiology, Addiriyah Chair for Environmental Studies, College of Science, King Saud University, P. O. Box 2455, Riyadh 11451, Saudi Arabia; 5 Visiting Professor Program (VPP), King Saud University, P.O. Box 2455, Riyadh 11451, Saudi Arabia

**Keywords:** Carotenoids, gene characterization, lutein, Scutellaria baicalensis, ß-carotene

## Abstract

*Scutellaria baicalensis *has a wide range of biological activities and has been considered as an important traditional drug in Asia and North America for centuries. A partial-length cDNA clone encoding phytoene synthase (SbPSY) and full-length cDNA clonesencoding phytoene desaturase (SbPDS), ξ-carotene desaturase (SbZDS), β-ring carotene hydroxylase (SbCHXB), and zeaxanthin epoxidase (SbZEP)were identifiedin *S. baicalensis*. Sequence analyses revealed that these proteins share high identity and conserved domains with their orthologous genes. *SbPSY*, *SbPDS*, *SbZDS*, *SbCHXB*, and *SbZEP* were constitutively expressed in the roots, stems, leaves, and flowers of *S*.*baicalensis*. *SbPSY*, *SbPDS*, and *SbZDS* were highly expressed in the stems, leaves, and flowers and showed low expression in the roots, where only trace amounts of carotenoids were detected. *SbCHXB* and *SbZEP* transcripts were expressed at relatively high levels in the roots, stems, and flowers and were expressed at low levels in the leaves, where carotenoids were mostly distributed. The predominant carotenoids in *S*.*baicalensis*were lutein and β-carotene, with abundant amounts found in the leaves (517.19 and 228.37 μg g^-1^ dry weight, respectively). Our study on the biosynthesis of carotenoids in *S. baicalensis* will provide basic data for elucidating the contribution of carotenoids to the considerable medicinal properties of *S. baicalensis*.

## Abbreviations

DEPC, diethylpyrocarbonate; HPLC, high-performance liquid chromatography; GGDP, geranylgeranyl diphosphate; PSY, phytoene synthase; PDS, phytoene desaturase; ZDS, ξ-carotene desaturase; LCYB, lycopene ß-cyclase; LCYE, lycopene ε-cyclase; CHXB, ß-ring carotene hydroxylase; CHXE, ε-ring carotene hydroxylase; ZEP, zeaxanthin epoxidase; CCD, carotenoid cleavage dioxygenase; NCED, 9-cis epoxycarotenoid dioxygenase; ABA, abscisic acid

## Introduction

Carotenoids are widely distributed in nature and represent the largest pigment group, with over 600 members identified to date (Cunningham and Gantt, 1998[5]). In plants, carotenoids play several important functions, such as the stabilization of lipid membranes (Havaux, 1998[10]), light collection for photosynthesis, and protection of the photosystem from photo-oxidation (Frank and Cogdell, 1996[7]; Ledford and Niyogi, 2005[19]). Carotenoids provide the yellow, orange, and red colors in flowers and fruits to attract pollinators and agents of seed dispersal (Howitt and Pogson, 2006[12]). Furthermore, carotenoids are precursors of apocarotenoids, which act as hormones, signaling compounds, chromophores, and scent or aroma constituents (Giuliano et al., 2003[9]). In animals and humans, carotenoids are essential nutrients and health-promoting compounds that are not synthesized *de novo* and must be acquired from the diet (Parker, 1996[23]). ß-Carotene is the primary dietary source of vitamin A, the deficiency of which leads to xerophthalmia, blindness, and premature death (Mayne, 1996[21]). Carotenoids also have strong antioxidant properties that may prevent degenerative diseases and reduce the risk of certain forms of cancer (Giovannucci, 1999[8]; Mayne, 1996[21]).

**Figure 1:**
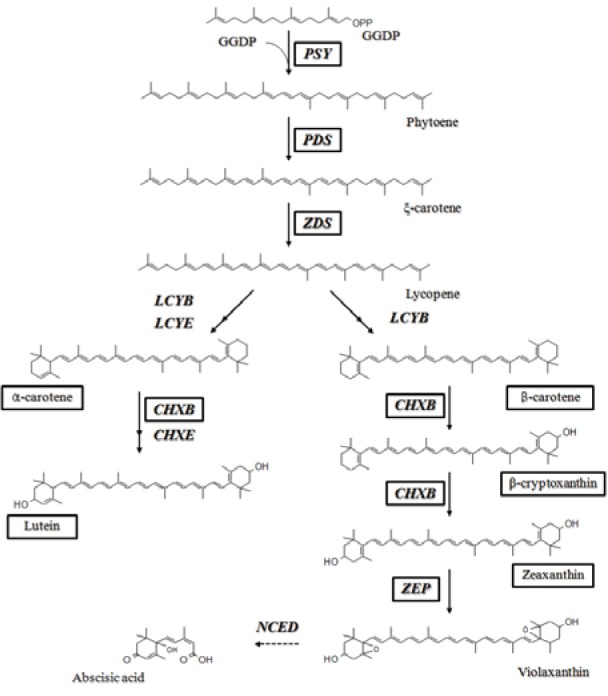
Carotenoid biosynthetic pathway in plants. The enzymes and carotenoids in black boxes were analyzed in this study. GGDP, geranylgeranyl diphosphate; PSY, phytoene synthase; PDS, phytoene desaturase; ZDS, ξ-carotene desaturase; LCYB, lycopene ß-cyclase; LCYE, lycopene ε-cyclase; CHXB, ß-ring carotene hydroxylase; CHXE, ε-ring carotene hydroxylase; ZEP, zeaxanthin epoxidase; NCED, 9-*cis* epoxycarotenoid dioxygenase

The carotenoid biosynthetic pathway has been extensively studied in higher plants (Cunningham and Gantt, 1998[5]). While carotenoids are synthesized in plastids, the corresponding genes are located in the nucleus, and their protein products are imported into the plastids. The most important step in the carotenoid biosynthetic pathway is the condensation of 2 geranylgeranyl diphosphate (GGDP) molecules to form phytoene; this process is catalyzed by phytoene synthase (PSY) (Figure 1(Fig. 1)). Phytoene undergoes a series of 4 desaturations to form lycopene via ξ-carotene, which is catalyzed by 2 enzymes, phytoene desaturase (PDS) and ξ-carotene desaturase (ZDS). The cyclization of lycopene is a branching point in the pathway: one leads to a-carotene and the other leads to ß-carotene. The formation of α-carotene requires the actions of lycopene ß-cyclase (LCYB) together with lycopene ε-cyclase (LCYE), and LCYB converts lycopene to ß-carotene in 2 reactions. Thereafter, α-carotene and ß-carotene are hydroxylated to produce lutein and zeaxanthin, respectively; this reaction is catalyzed by ß-ring carotene hydroxylase (CHXB) and ε-ring carotene hydroxylase (CHXE). Further epoxidation of zeaxanthin by zeaxanthin epoxidase produces violaxanthin, which is used to synthesize plant hormone abscisic acid (ABA) through oxidative cleavage catalyzed by 9-cis epoxycarotenoid dioxygenase (NCED) (Schwartz et al., 1997[27]). Elucidation of these biosynthetic molecules has increased our understanding of the mechanisms through which carotenoids function.

*Scutellaria baicalensis *has been used as an important traditional drug for the treatment of dysentery, pyrexia, jaundice, and carbuncles in Asia and North America for centuries (Joshee et al., 2002[15]; Tang and Eisenbrand, 1992[28]). Pharmacological reports have indicated that S. baicalensis has a multitude of medicinal properties, including anti-inflammatory, antidiabetic, antiviral, antihypertension, antioxidant, and anticancer effects (Chan et al., 2000[4]; Huang et al., 2006[14]; Waisundara et al., 2008[33]). The biological activities of S. baicalensis are likely to be related to the variety of flavones, phenylethanoids, amino acids, sterols and essential oils found in this plant (Joshee et al., 2002[15]; Tang and Eisenbrand, 1992[28]). However, except for flavonoids, which are believed to be the major component of *S. baicalensis*, very few studies have described the health benefits of other compounds of* S. baicalensis*.

The goal of this study was to investigate the relationship between the expression levels of carotenoid biosynthetic genes and carotenoid accumulation in* S. baicalensis*. Here, partial-length cDNA encoding PSY and full-length cDNAs encoding PDS, ZDS, CHXB, and ZEP were isolated. Furthermore, transcript levels of carotenoid biosynthetic genes and carotenoid accumulation were analyzed in different organs of *S. baicalensis* using real-time PCR (qRT-PCR) and high performance liquid chromatography (HPLC), respectively.

## Materials and Methods

### Plant materials

*S. baicalensis* Georgi plants were grown under greenhouse conditions for 1 month and then transferred to the experimental farm of Chungnam National University (Daejeon, Korea). After flowering, the plants were dissected into the roots, stems, leaves, and flowers. All samples were immediately frozen in liquid nitrogen and then stored at 80 °C and/or freeze-dried for RNA isolation and HPLC analysis.

### RNA isolation and cDNA synthesis

The samples were ground in a mortar with liquid nitrogen and total RNA was extracted from frozen powder using a Plant Total RNA Mini Kit (Geneaid, Taiwan) according to the manufacturer’s instructions. Gel electrophoresis and spectrophotometer were performed to test the quality and concentration of total extracted RNA, respectively. For first-strand cDNA synthesis, 1 µg of high-quality total RNA was used for reverse transcription using the ReverTra Ace-R kit (Toyobo, Japan). A 20-fold dilution of the 20 µL resulting cDNA was used as template qRT- PCR.

### Isolation of cDNAs encoding enzymes involved in carotenoid biosynthetic pathway

In another study (unpublished data), we obtained 39,581 different genes from *S. baicalensis* using next generation DNA sequencing platforms (Roche/454 GS_FLX+ and Illumina/Solexa HiSeq2000). A partial-length cDNA encoding PSY and full-length cDNAs encoding PDS, ZDS, CHXB, and ZEP were indentified from this database. These proteins were then analyzed for homologies with known sequences and designed as SbPSY, SbPDS, SbZDS, SbCHXB, and SbZEP (GenBank accession numbers: KC417312, KC417313, KC417314, KC417315, and KC417316, respectively).

### Sequence analysis

The deduced amino acid sequences of carotenoid biosynthetic genes from *S. baicalensis* were analyzed for homology using the BLAST program at the NCBI GenBank database (http://www.ncbi.nlm.nih.gov/ BLAST). Sequence alignments were carried out using BioEdit Sequence Alignment Editor, version 5.0.9 (Department of Microbiology, North Carolina State University, Raleigh, NC, USA). The predicted molecular mass of protein was calculated by online website (http://www.sciencegateway.org/ tools/proteinmw.htm).

### Quantitative real-time PCR

QRT-PCR primers amplified five target carotenoid biosynthetic genes and Actin housekeeping gene (HQ847728) of *S. baicalensis* were designed using the Primer3 website (http://frodo.wi.mit.edu/primer3/) (Table 1(Tab. 1)) and then tested for the specificity by PCR. For quantification of standard, the PCR products amplified from cDNA were purified, and the concentration of the products was measured to calculate the number of cDNA copies. The expression of five carotenoid biosynthetic genes was calculated by the method of relative quantification to Actin as the reference. Real-time PCR reactions were carried out in a 20-µl reaction mix containing 5 µl of template cDNA, 10 µl of 2× SYBR Green Realtime PCR Master Mix (Toyobo, Japan), 0.5 µl of each primer (10 µM), and DEPC water. Thermal cycling conditions were as follows: 95 °C for 5 min; 40 cycles of 95 °C for 15 s, 56 °C for 15 s, 72 °C for 20 s. PCR products were analyzed using Bio-Rad CFX Manager 2.0 software. Three replications for each sample were used for real-time analysis.

### Carotenoid extraction and HPLC analysis

Extraction and measurement of carotenoids by high-performance liquid chromatography (HPLC) were performed as described by our group (Kim et al., 2012[16]). Briefly, carotenoids were released from the *S. baicalensis* samples (0.02 g) by adding 3 mL of ethanol containing 0.1 % ascorbic acid (w/v), vortex mixing for 20 s and placing in a water bath at 85 °C for 5 min. The carotenoid extract was saponified with potassium hydroxide (120 µL, 80 % w/v) at the 85 °C water bath for 10 min. After saponification, samples were placed immediately on ice, and cold deionised water (1.5 mL) was added. ß-Apo-8'-carotenal (0.2 mL, 25 g/mL) was added as an internal standard. Carotenoids were extracted twice with hexane (1.5 mL) by centrifugation at 1,200 × g to separate the layers. Aliquots of the extracts were dried under a stream of nitrogen and re-dissolved in 50:50 (v/v) dichloromethane/methanol before analysis by HPLC. The carotenoids were separated on a C30 YMC column (250 × 4.6 mm, 3 µm; Waters Corporation, Milford, MA, USA) by Agilent 1100 HPLC (Massy, France) equipped with a photodiode array (PDA) detector. Chromatograms were generated at 450 nm. Solvent A consisted of methanol/water (92:8 v/v) with 10 mM ammonium acetate. Solvent B consisted of 100 % methyl tert-butyl ether. Gradient elution was performed at 1 mL/min under the following conditions: 0 min, 90 % A/10 % B; 20 min, 83 % A/17 % B; 29 min, 75 % A/25 % B; 35 min, 30 % A/70 % B; 40 min, 30 % A/70 % B; 42 min, 25 % A/75 % B; 45 min, 90 % A/10 % B; and 55 min, 90 % A/10 % B. Carotenoid standards were purchased from CaroteNature (Lupsingen, Switzerland). For quantification purpose, calibration curves were drawn by plotting at four different concentrations of carotenoid standards according to the peak area ratios with ß-apo-8'-carotenal. 

## Results and Discussion

### Sequence analyses of carotenoid biosynthetic genes from S. baicalensis

**Figure 2:**
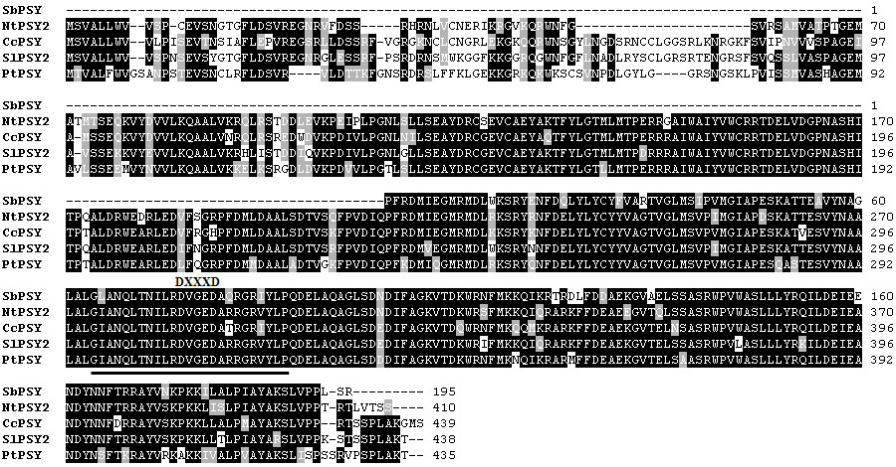
Multiple alignments of the amino acid sequences of SbPSY with other PSYs. Identical residues are indicated by a black background, and similar residues are shaded with a gray background. The solid underline is the trans-IPPS-HH domain. DXXXD, where X encodes for any amino acid, represents the aspartate-rich region. NtPSY2,* Nicotiana tabacum *(JX101474); CcPSY, *Coffea canephora* (DQ157164); SlPSY2, *Solanum lycopersicum* (NM_001247742); PtPSY, *Populus* *trichocarpa* (XM_002327528).

SbPSY was composed of 585 bp encoding a partial open reading frame (ORF) of 195 amino acids. A BLAST search at the amino acid level showed that SbPSY exhibited high homology to other PSYs (Figure 2(Fig. 2)). Specifically, SbPSY shared 86 % identity and 95 % similarity with* Nicotiana tabacum* PSY, 86 % identity and 93 % similarity with *Coffea canephora* PSY, 86 % identity and 93 % similarity with* Solanum lycopersicum* PSY, and 83 % identity and 93 % similarity with *Populus trichocarpa* PSY. The trans-isoprenyl diphosphate synthase, for the head-to-head condensation reaction (trans-IPPS-HH) domain and aspartate-rich region (DXXXD, where X encodes any amino acid) conserved in other PSY genes were found in SbPSY (Ohnuma et al., 1996[22]; Rasid et al., 2008[24]).

**Figure 3:**
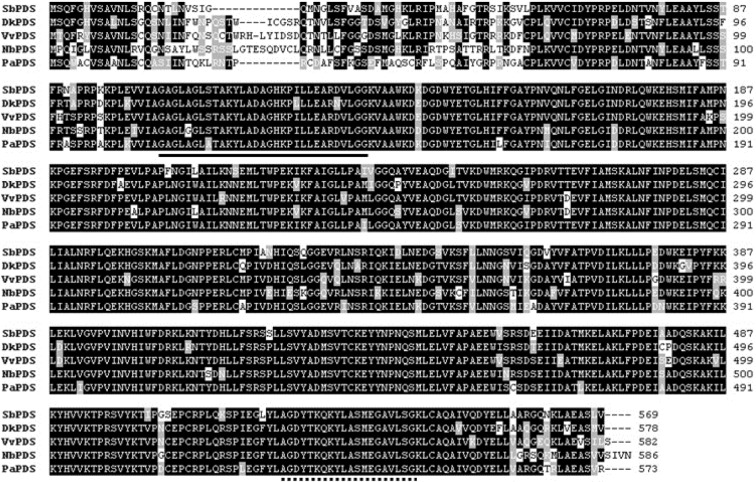
Multiple alignments of the amino acid sequences of SbPDS with other PDSs. Identical residues are indicated by a black background, and similar residues are shaded with a gray background. The solid underline indicates the dinucleotide-binding domain, and the dotted underline indicates the carotenoid-binding domain. DkPDS, *Diospyros kaki *(GU112527); VvPDS, *Vitis vinifera* (XM_002264231); NbPDS, *Nicotiana* *benthamiana* (DQ469932); PaPDS, *Prunus armeniaca* (AY822065).

SbPDS was 2348 bp long and had an ORF of 1710, encoding a protein of 569 amino acids with a predicted molecular mass of 63.38 kDa (Figure 3(Fig. 3)). SbPDS was 85 %, 84 %, 83 %, and 83 % identical to PDS from *Diospyros kaki*, *Vitis vinifera*, *N. benthamiana*, and *Prunus armeniaca*, respectively. As shown in Figure 3(Fig. 3), SbPDS contained a conserved dinucleotide-binding motif (GXGX2GX3AX2LX3GX6EX5GG) and a carotenoid-binding domain also found in other orthologous genes (Yan et al., 2011[34]; Zhu et al., 2005[35]).

**Figure 4:**
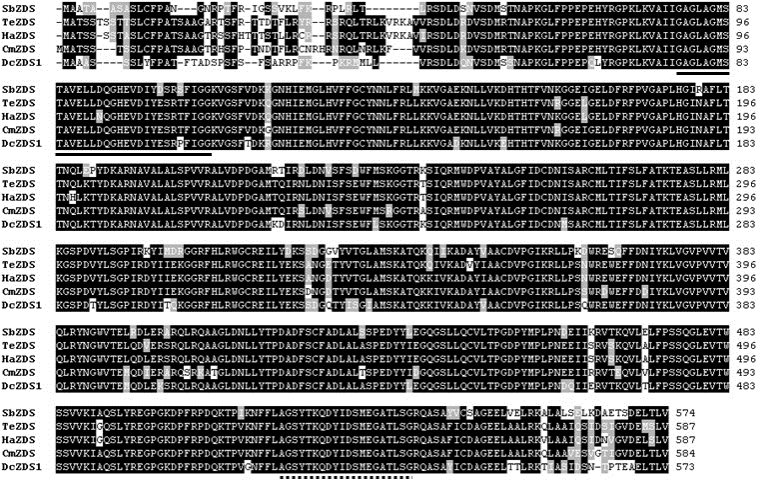
Multiple alignments of the amino acid sequences of SbZDS with other ZDSs. Identical residues are indicated by a black background, and similar residues are shaded with a gray background. The solid underline indicates the dinucleotide-binding domain, and the dotted underline indicates the carotenoid-binding domain. TeZDS, *Tagetes erecta* (AF251013); HaZDS, *Helianthus annuus* (AJ438587); CmZDS, *Chrysanthemum x morifolium* (AB205052); DcZDS1, *Daucus carota* (DQ222430).

SbZDS was composed of 2159 bp, with a 1725-bp ORF encoding a protein of 574 amino acids (predicted molecular mass of 63.55 kDa; Figure 4(Fig. 4)). The closest homolog of SbZDS was ZDS from* Tagetes erecta* (85 % identity and 91 % similarity) followed by ZDS from *Helianthus annuus* (84 % identity and 91 % similarity), ZDS from *Chrysanthemum x morifolium* (83 % identity and 90 % similarity), and ZDS from *Daucus carota *(84 % identity and 91 % similarity). Similar to SbPDS, SbZDS also contained a conserved dinucleotide-binding motif at the N-terminus and a carotenoid-binding domain at the C-terminus (Figure 4(Fig. 4)).

**Figure 5:**
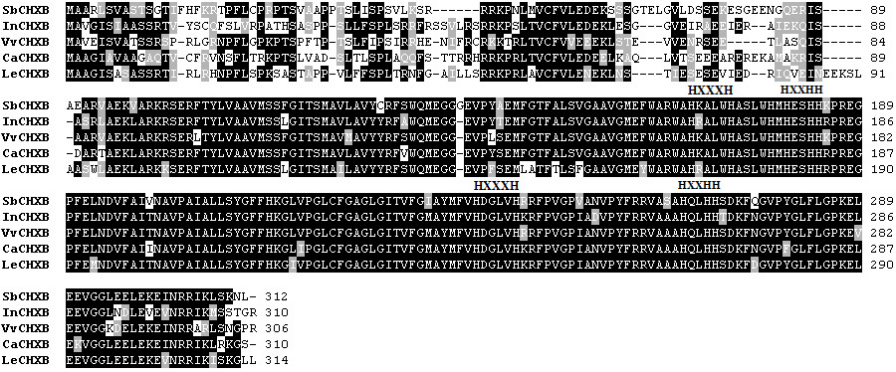
Multiple alignments of the amino acid sequences of SbCHXB with other CHXBs. Identical residues are indicated by a black background, and similar residues are shaded with a gray background. HXXXH and HXXHH, where X encodes for any amino acid, represent 4 conservatively spaced histidine motifs. InCHXB, *Ipomoea nil *(AB499058); VvCHXB,* Vitis vinifera* (XM_002273545); CaCHXB, *Coffea arabica* (DQ157169); LeCHXB, *Lycopersicon esculentum* (DQ864755).

SbCHXB consisted of 1410 bp, with a 939-bp ORF encoding a protein of 312 amino acids (predicted molecular mass of 34.48 kDa; Figure 5(Fig. 5)). SbCHXB exhibited 75 % identity and 84 % similarity with* Ipomoea nil* CHXB, 76 % identity and 83 % similarity with *Vitis vinifera* CHXB, 78 % identity and 85 % similarity with *C. arabica* CHXB, and 74 % identity and 82 % similarity with *Lycopersicon esculentum* CHXB. Four conservatively spaced histidine motifs proposed to be involved in iron binding during hydroxylation reactions are presented in Figure 5(Fig. 5) (Bouvier et al., 1998[3]). 

**Figure 6:**
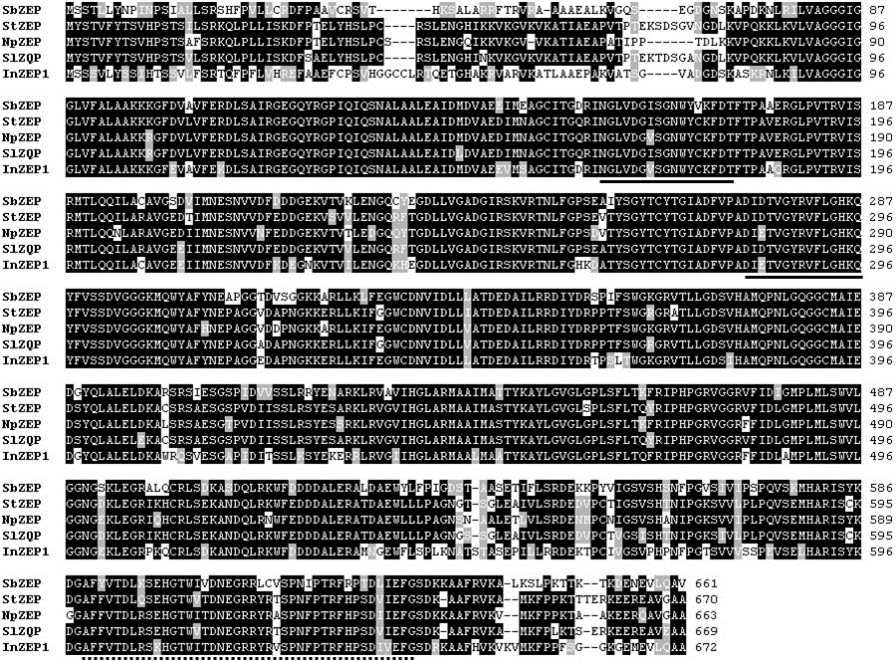
Multiple alignments of the amino acid sequences of SbZEP with other ZEPs. Identical residues are indicated by a black background, and similar residues are shaded with a gray background. The solid underline indicates the short motifs typical of the lipocalin family of proteins, and the dotted underline indicates the FHA domain. StZEP, *Solanum tuberosum* (DQ206629); NpZEP, *Nicotiana plumbaginifolia* (X95732); SlZEP,* Solanum lycopersicum* (EU004202); InZEP1, *Ipomoea nil *(HQ827173).

SbZEP was composed 2399 bp, with an ORF of 1986 bp encoding a protein of 661 amino acids with a predicted molecular mass of 72.09 kDa (Figure 6(Fig. 6)). SbZEP shared 76 % identity and 84 % similarity with *Solanum tuberosum* ZEP, 76 % identity and 83 % similarity with *N. plumbaginifolia* ZEP, 76 % identity and 84 % similarity with *S. lycopersicum* ZEP, and 75 % identity and 84 % similarity with *Ipomoea nil* ZEP. SbZEP displayed 2 short motifs typical of the lipocalin family of proteins and a phosphopeptide-binding domain (FHA domain), which are likely to be the common features of ZEP genes (Durocher and Jackson, 2002[6]; Hieber et al., 2000[11]).

### Expression levels of carotenoid biosynthetic genes in different organs of S. baicalensis

**Figure 7:**
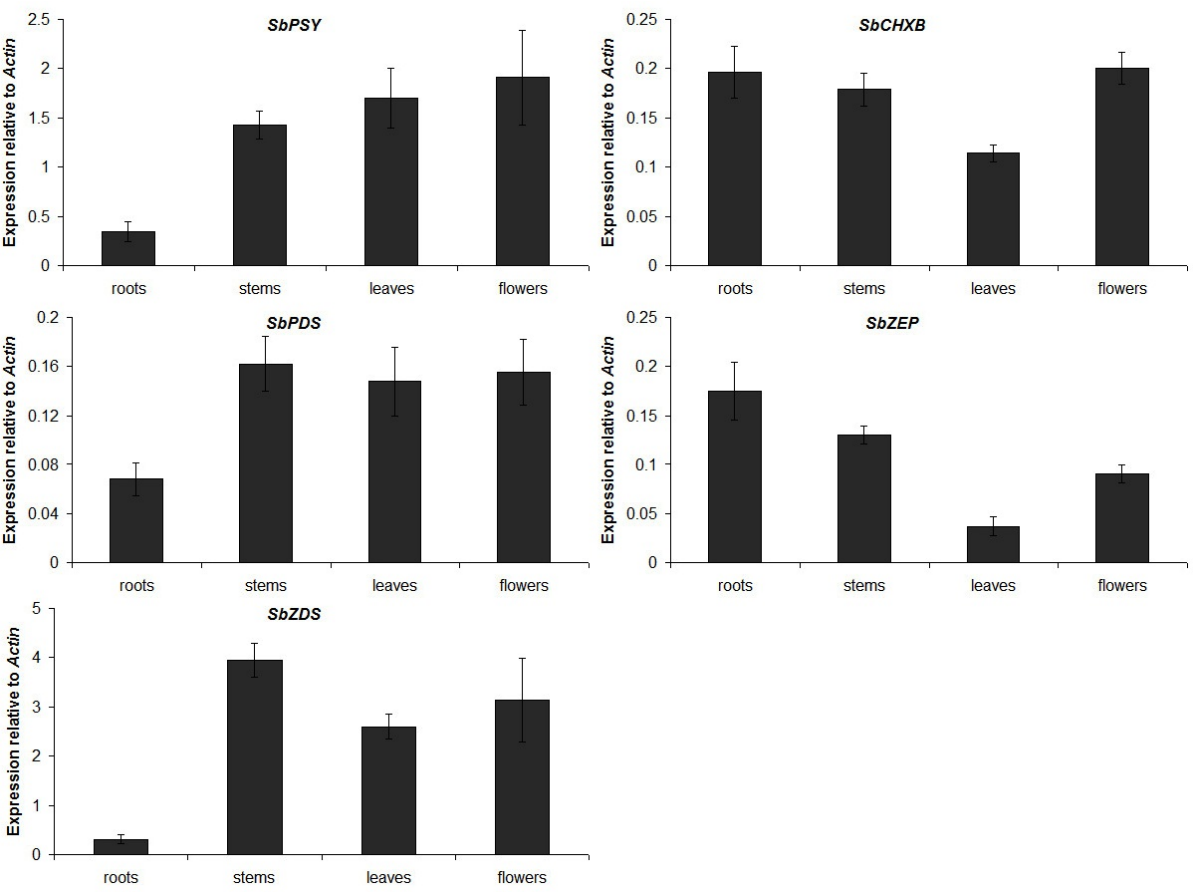
Expression levels of carotenoid biosynthetic genes in different organs of *S. baicalensis*. The height of each bar and the error bars show the mean and standard error, respectively, from 3 independent measurements.

The expression of carotenoid biosynthetic genes was investigated in the roots, stems, leaves, and flowers of *S. baicalensis* by real-time PCR (Figure 7(Fig. 7)). One of the initial proteins involved in the carotenoid biosynthetic pathway, SbPSY, was expressed at the highest levels in the flowers and was found at lower levels in the leaves and stems, with the lowest expression observed in the roots. Similar to SbPSY, the expression levels of SbPDS and SbZDS were low in the roots and relatively high in the stems, leaves, and flowers. SbCHXB and SbZEP showed different transcriptional patterns with the first 3 enzymes in the carotenoid biosynthetic pathway. The mRNA transcript levels of SbCHXB and SbZEP were lowest in the leaves and higher in the roots, stems, and flowers.

### Analysis of carotenoid content in different organs of S. baicalensis

The same plant materials as those used for qRT-PCR were used to analyze the composition and content of carotenoids in *S. baicalensis* using HPLC (Table 2(Tab. 2)). In general, carotenoids were distributed mostly in the leaves, and the majority of carotenoids detected in *S. baicalensis* were lutein and ß-carotene. Specifically, highly abundant lutein content (517.19 µg g^-1^) was found in the leaves, whereas only a small amount of its precursor, α-carotene (2.23 µg g^-1^), was found in the same organ. The leaves also contained a significant amount of ß-carotene (228.37 µg g^-1^), which may explain the presence of considerable amounts of its *cis* isomers, 9-*cis* ß-carotene (21.36 µg g^-1^) and 13-*cis* ß-carotene (29.72 µg g^-1^). In contrast, accumulation of ß-carotene may cause poor accumulation of its derivatives, ß-cryptoxanthin (1.93 µg g^-1^) and zeaxanthin (1.94 µg g^-1^). In the stems, lutein (43.16 µg g^-1^) and ß-carotene (14.08 µg g^-1^) were also the major carotenoids. Only miniscule amounts of 9-*cis* ß-carotene (2.08 µg g^-1^), 13-*cis* ß-carotene (1.12 µg g^-1^), ß-cryptoxanthin (0.33 µg g^-1^), zeaxanthin (0.85 µg g^-1^), and no α-carotene were measured in the stems. The flowers accumulated lower amounts of lutein (13.18 µg g-1) and ß-carotene (6.99 µg g^-1^) than the stems. Other carotenoids were either expressed at very low levels or were absent in the flowers. In the roots, only trace amounts of lutein (0.88 µg g^-1^) and ß-carotene (0.35 µg g^-1^) could be found.

In the present study, a partial-length cDNA encoding PSY and full-length cDNA encoding PDS, ZDS, CHXB, and ZEP were characterized, and carotenoid contents were analyzed in *S. baicalensis*. Carotenoid biosynthetic genes were constitutively expressed in all examined organs of *S. baicalensis* with different patterns between upstream genes and downstream genes. The transcription levels of *SbPSY*, *SbPDS*, and *SbZDS* were high in the stems, leaves, and flowers and low in the roots, while the transcription levels of *SbCHXB* and *SbZDS* were low in the leaves and relatively high in other organs. PSY, which catalyzes the first committed and rate-limiting step in carotenoid biosynthesis, is the major key regulator of carotenoid accumulation in plants (Rodríguez-Villalón et al., 2009[25]; Toledo-Ortiz et al., 2010[29]), suggesting that the low expression levels of *SbPSY*, *SbPDS*, and *SbZDS* caused only trace amounts of carotenoids to be found in the roots of* S. baicalensis*. However, *SbPSY*, *SbPDS*, and *SbZDS* were highly expressed in the stems and flowers, where only small amounts of carotenoids were detected. Further along in the pathway, carotenoids can be cleaved at various chain positions by carotenoid cleavage dioxygenases (CCDs) to form a broad range of apocarotenoids (Auldridge et al., 2006[2]; Huang et al., 2009[13]; Kloer and Schulz, 2006[17]). An inverse correlation between CCD expression and carotenoid content was observed in *Arabidopsis* (Auldridge et al., 2006[1]) and bitter melon (Tuan and Park, 2013[32]). Therefore, we hypothesize that the small amounts of carotenoids in the flowers and stems may be due to the high expression of CCDs. Further studies on CCDs in *S. baicalensis* are needed to clarify this hypothesis.

In the ß,ß-carotenoid branch, ß-carotene is hydroxylated by CHXB to produce zea-xanthin, which is then converted into viola-xanthin by ZEP. Low expression levels of *SbCHXB* and *SbZEP* probably result in the large accumulation of ß-carotene in the leaves of *S. baicalensis*. Unlike the findings for the ß,ß-carotenoid branch, in the ε,ß-carotenoid branch, lutein content was most abundant in the leaves, where its biosynthetic enzyme, *SbCHXB*, was expressed in low amounts. Indeed, *SbCHXB* has been proposed to have an active role in the flux of the ß,ß-carotenoid branch rather than the ε,ß-carotenoid branch of carotenoid biosynthesis in* S. baicalensis*.

Carotenoids are rarely biosynthesized in the underground organs (roots), but are very abundant in the photosynthetic organs (leaves) of* S. baicalensis*. This mechanism is similar to that in other plants, such as garlic (Tuan et al., 2011[30]) and bitter melon (Tuan et al., 2011[31]), and indicates the essential role of light in the accumulation of carotenoids in plants. Furthermore, we discovered that besides the roots (the most popular part of *S. baicalensis*), the leaves, which contain significant amounts of lutein (517.19 µg g^-1^) and ß-carotene (228.37 µg g^-1^), are also a potential medicinal part of this plant. For humans, lutein is believed to function as an important antioxidant and anti-inflammatory (Ma and Lin, 2010[20]; Sabour-Pickett et al., 2012[26]), and ß-carotene has been shown to have the capacity to reduce the risk of stroke, heart disease, and cancer (Kritchevsky, 1999[18]; Mayne, 1996[21]).

In conclusion, the molecular characterization of carotenoid biosynthetic genes along with carotenoid content may be useful to clarify the biosynthetic mechanisms of carotenoids in *S. baicalensis* in particular and in plants in general. In addition, these data will provide a foundation for the elucidation of the contribution of carotenoids to the great medicinal properties of *S. baicalensis*.

## Notes

Pham Anh Tuan and Yeon Bok Kim have contributed equally to this work.

Naif Abdullah Al-Dhabi and Sang Un Park have contributed equally as corresponding authors to this work.

## Acknowledgements

This project was supported by King Saud University, Deanship of Scientific Research, Addiriyah Chair for Environmental Studies.

## Figures and Tables

**Table 1:**
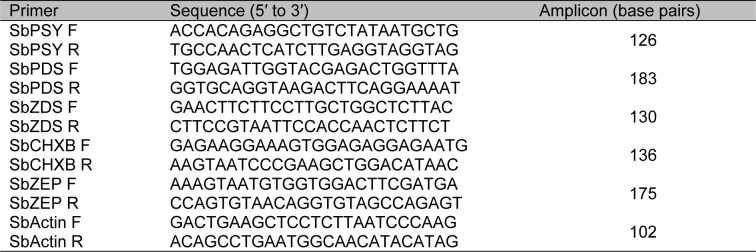
Primers used for real-time PCR

**Table 2:**
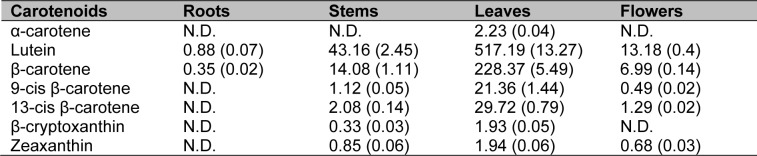
Carotenoid composition and content in different organs of *S. baicalensis* (µg g^-1^ dry weight). The results have been expressed as mean (± standard errors of the mean) values (n = 3). N.D. = not detected
